# Revision of reversed total shoulder arthroplasty. Indications and outcome

**DOI:** 10.1186/1471-2474-13-160

**Published:** 2012-08-27

**Authors:** Mazda Farshad, Marion Grögli, Sabrina Catanzaro, Christian Gerber

**Affiliations:** 1 Balgrist University Hospital, University of Zürich, Zürich, Switzerland

## Abstract

**Background:**

The complications of reversed total shoulder arthroplasty (RTSA) requiring an additional intervention, their treatment options and outcome are poorly known. It was therefore the purpose of this retrospective study, to identify the reasons for revision of RTSA and to report outcomes.

**Methods:**

Four hundred and forty-one performed RTSA implanted between 1999 and 2008 were screened. Sixty-seven of these cases had an additional intervention to treat a complication. Causes were identified in these 67 cases and the outcome of the first 37 patients who could be followed for more than two years after their first additional intervention was analyzed.

**Results:**

Of 441 RTSA, 67 cases (15%) needed at least one additional intervention to treat a complication, 30 of them needed a second, eleven a third and four a fourth additional intervention. The most common complication requiring a first intervention was instability (18%) followed by hematoma or superficial wound complications (15%) and complications of the glenoid component (12%). Patients benefitted from RTSA despite the need of additional interventions as indicated by a mean increase in total Constant-Murley score from 23 points before RTSA to 46 points at final follow-up (p < 0.0001).

**Conclusions:**

Instability, hematoma or superficial wound complications and complications of the glenoid component are the most common reasons for an additional intervention after RTSA. Patients undergoing an additional intervention as treatment of these complications profit significantly as long as the prosthesis remains in place.

## Background

Reverse total shoulder arthroplasties (RTSA) has become a valuable treatment option for irreparable rotator cuff failure [1-6], associated or not with osteoarthritis or prosthetic humeral replacement. The reversal of the physiological ball and socket configuration of the humerus and glenoid has become a clinically successful concept which, however, changes joint physiology and biomechanics [2,7] and bears the potential for complications which have been reported to be more frequent than those of conventional total shoulder replacement. Complications reported to be frequent are scapular notching, infection, instability, acromial fractures, hematoma formation and complications of the glenoid component [2,8-17]. The frequency of these complications has been described with variable accuracy of reporting and heterogeneity of methodology between the studies [18]. There is lack of information about clinical implications of at least some of these complications about treatment and eventual outcomes.

It was therefore the purpose of this retrospective study to identify and analyze the reasons for additional interventions needed for treatment of complications after RTSA and to report outcomes.

## Methods

Approval for retrospective analysis of data used in this study was given by the state ethical committee of Zürich, Switzerland. The institutional registry for shoulder and elbow prosthesis was screened to identify 480 consecutively implanted RTSA (232 Delta III™, Depuy and 248 Anatomical Reverse™, Zimmer) implanted between 1999 and 2008. Of those, 39 were either implantation of components of RTSA or complete RTSAs for prosthetic revisions leaving 441 RTSAs implanted for reasons other than prosthetic revision. A total of 96 patients with revision after RTSA were identified. Of those, the primary RTSA was performed in our institution in 67 patients (Revision rate = 15%) and 29 patients had been referred for treatment of complications after RTSA. Those 29 were excluded from further analysis due to lack of primary data and potential heterogeneity of implantation and postoperative treatment regimens. Fifty-five of the 67 patients had a minimum follow-up of two years following RTSA. Thirty-seven of the 55 patients had a minimum follow-up of two years after their first revision. The charts including all operative protocols of all 67 cases were retrospectively analyzed to identify basic patients demographics, the number and type of additional interventions performed in each patient, reasons for each additional intervention and the performed procedure for all included patients. For assessment of outcome, the 37 patients with a follow-up of more than 2 years after the additional intervention were contacted and the clinical outcome, including physical and radiographic examination and assessment of Subjective Shoulder Value (SSV) and Constant-Murley score [19] could be obtained in 31 patients. Six patients could not be personally reviewed and the Constant-Murley score could not be documented at follow-up: two patients had died from unrelated causes, one patient had pursued treatment in another institution and three patients could not be reviewed because two institutionalized for dementia were unable to respond and one could not be traced. A subgroup was formed to analyze patients outcomes with the most common indications for an additional intervention, namely instability.

### Statistical analysis

Data were statistically tested for normal distribution using the D’Agostino and Pearson omnibus normality test before applying either two tailed paired Students-t-test or Wilcoxon test for normally and not-normally distributed data for intra-sample comparison. Values are reported as mean and standard deviations or 95% confidence interval where appropriate. All statistical tests were performed using a commercial statistical software package, with significance levels set at p < 0.05.

## Results

### Reasons for revision after RTSA

Baseline characteristics are summarized in Table 1. Sixty-seven patients with a mean age of 69 ± 9 years (male to female ratio of 0.5 to 1) needed at least one additional intervention to treat a complication of RTSA, thirty of them needed a second, eleven a third and four a forth intervention. Additional interventions after RTSA were needed at between the same day of the RTSA and eight years thereafter, with a mean of 17 months postoperatively. Overall, the most common reason for the first intervention was instability resulting in subluxation or dislocation (n = 12, (18%)) occurring at a mean of 17 months after initial RTSA (5 days to 72 months). However this complication was seen in 11 of 44 shoulders with Delta-III prostheses and in 1 of 23 patients with Anatomical Reverse prostheses (significant difference with confidence of >90% by a two tailed z-test of proportions). It was definitely treated by closed reduction in those five cases if no further component re-dislocation was radiologically evident and no recurrent subluxation was reported. A change of components of the prosthesis was necessary in seven cases: Selection of a higher humeral polyethylene inlay was used in three, revision of a glenoid component in two and revision of a humeral component in two cases. The second most common reasons for revision were hematoma and superficial wound problems (e.g. dehiscence) (n = 10, (15%)) occurring after a mean of 38 days. Hematoma was treated by simple evacuation in seven cases. Other frequent reasons for revision were complications of the glenoid component (n = 8, (12%)) and acromial (n = 5) or coracoid fractures (n = 2, (10%)). Complications of the glenoid component were treated in four cases by change of the glenoid (1 Delta and 3 Anatomical) component and in three cases by conversion (1 Delta and 2 Anatomical) to a hemi-prosthesis. In one case infection was suspected and a spacer was implanted (Table 2). Infections, deficiency in external rotation, acromio-clavicular pathologies and humeral component loosening were followed by other infrequent reasons such as wear of components, metallosis and other rare events (Table 3). The two most common reasons for a second intervention were instability (n = 7, 23% of all reasons for the second intervention) and infections (n = 6, 20%). The most common reason for the third (n = 5, 45%) and forth (n = 2, 50%) intervention was again instability.

**Table 1:** Basic characteristics and indication for primary RTSA in 67 patients with revisions after RTSA

**Basic charactersitics**	**Total (n = 67)**	**Delta III (n = 44)**	**Anatomical Reverse (n = 23)**
age	69 ± 9 years	69 ± 9 years	69 ± 10 years
male : female	23 : 44	16:28	7:16
right : left	39 : 28	27:17	12:11
dominant arm	42 of 67	27 of 44	15 of 23
**Indications for primary RTSA**			
RCR and OA	24	16	8
Irreparable RCR	17	13	4
Failed hemiarthroplasty	16	9	7
Sequels of fracture	10	6	4

RCR: Rotator cuff rupture, OA: Osteoarthritis.

**Table 2:** Complications of the glenoid component as reason for the first re-intervention after RTSA

	**Primary Indication for RTSA**	**Complication leading to re-intervention**	**Treatment of the complications**
	(n=)	(n=)	(n=)
**Anatomical Reverse**	Irreparable RCR (2)	Glenoid loosening (2)	Glenoid change (2)
	Irreparable RCR and OA (3)	Pullouts (3)	Conversions to hemi-TP (2)
			Spacer implantation (1)
**Delta III**	Irreparable RCR (1)	Glenoid loosening (1)	Glenoid change (2)
	Failed of hemi-prosthesis (1)	Pullout (1)	Conversion to hemi-prosthesis (1)
	Sequel of fracture (1)	Glenosphere disassembly (1)	

RCR: Rotator cuff rupture, OA: Osteoarthritis.

**Table 3:** Reasons for the first re-intervention after RTSA

	**Total**		**Delta III**		**Anatomical Reverse**	
	(n = 67)	*%*	(n = 44)	%	(n = 23)	%
Instability*	12	*17.9*	11	*25.0*	1	*4.3*
Wound dehiscence/ hematoma	10	*14.9*	8	*18.2*	2	*8.7*
Complications of the glenoid component	8	*11.9*	3	*6.8*	5	*21.7*
Acromial or coracoid fracture	7	*10.4*	5	*11.4*	2	*8.7*
Pain*	6	*8.9*	1	*2.3*	5	*21.7*
Deficient external rotation	5	*7.5*	2	*4.5*	3	*13.0*
Infection	3	*4.5*	3	*6.8*	0	*0.0*
Humeral component loosening	3	*4.5*	3	*6.8*	0	*0.0*
Disturbing ORIF material	2	*3.0*	2	*4.5*	0	*0.0*
Periprosthetic fracture	2	*3.0*	2	*4.5*	0	*0.0*
Dislocation of greater tuberosity	2	*3.0*	1	*2.3*	1	*4.3*
Component wear	2	*3.0*	2	*4.5*	0	*0.0*
Deficient internal rotation	1	*1.5*	0	*0.0*	1	*4.3*
Subacromial impingement	1	*1.5*	0	*0.0*	1	*4.3*
Heterotopic ossification	1	*1.5*	1	*2.3*	0	*0.0*
Metallosis (inferior screw notching)	1	*1.5*	0	*0.0*	1	*4.3*
Humeral component subsidence	1	*1.5*	0	*0.0*	1	*4.3*

*Significantly different (Delta-III vs. Anatomical Reverse).

### Outcome after revision of RTSA

Thirty-seven patients could be reviewed at no less than two years with a mean follow-up of 47 (range 24 to 150) months after the first revision. Twenty-one of these 37 patients needed a second, nine a third and four a forth additional intervention to treat complications associated with RTSA. The most common cause for an re-intervention in these 37 patients was similar to the total cohort of 67 patients. Instability (n = 9, (24%)) was the most common reason for an intervention followed by hematoma or superficial wound complications (n = 6, (16%)) and complications of the glenoid component (n = 3, (8%)). However, an external rotation deficit perceived as disabling and correctable was also a relevant reason for revision in this subgroup (n = 5, (14%)). The most common indication of an additional intervention consistently was instability in the second (n = 5, (24% of all revision causes)), third (n = 4, (44%)) and forth additional intervention (n = 2, 50%)). The total Constant-Murley score of the 31 personally examined patients increased by 24 points (95% CI: 17, 31) (p < 0.0001) from the time-point immediately before implantation of the RTSA to the last follow-up at an mean of 49 months (95% CI: 1382, 1777 days) after RTSA. There was a significant increase in SSV, relative Constant-Murley score, abduction strength and a substantial decrease in pain (Table 4). There was an increase in ability to abduct the arm, but a loss of active internal rotation (Table 4).

**Table 4:** Clinical outcome of patients with a minimal follow-up of 2 years after the first re-intervention (n = 31) after RTSA

	**Before RTSA**		**Last follow-up**		
	*mean*	*(95% CI)*	*mean*	*(95% CI)*	
Total Constant-Murley score	23	(17.8, 27.2)	46	(38.0, 53.8)	*p < 0.0001*
Relative Constant-Murley score (%)	30	(23.9, 36.8)	62	(51.8, 73.0)	*p < 0.0001*
Subjective Shoulder Value (%)	18	(11.2, 24.2)	49	(39.9, 57.2)	*p < 0.0001*
Pain (points as in Constant-Murley score)	5.4	(3.87, 6.9)	11	(9.4, 12.7)	*p < 0.0001*
Abduction strength (kg)	0.29	(0, 0.63)	1.9	(0.97, 2.82)	*p < 0.001*
Range of motion:					
Abduction (°)	49	(35.9, 62.1)	97	(81.1, 112.8)	*p < 0.0001*
Internal rotation (°)	41	(29.9, 52)	14	(3.9, 24.8)	*p < 0.001*
External rotation (°)	5.5	(−5.0, 16.0)	10.8	(0.6, 21.0)	*p = 0.2757*

### Instability and treatment procedure

The indication for the first closed or open revision was instability with twelve of 67 cases. These twelve patients did not differ from the entire cohort of 67 patients with regard to age (70 ± 7 years vs 69 ± 9 years) or gender (male to female: 0.5 to 1 in both groups) but to the type of prosthesis used for primary RTSA (Delta to Anatomical: 11:1 compared to 44:23). Early occurrence of instability (mean 101 days) was treated by closed reduction under general anesthesia (n = 5), later occurrence of instability (mean 781 days) was treated operatively (n = 7). From those five patients who were treated with closed reduction initially, in two patients a second intervention was performed subsequently; one was treated with change of the inlay (two days after reposition) and the other patient needed a closed reduction one day after initial reposition and a change of the humeral component was performed 3 weeks after.

In the other seven patients in whom instability occurred later, lengthening the prosthesis using a thicker inlay was performed in three cases, a change of the glenoid component in two and exchange of the humeral component in other two cases. One patient in whom the glenoid component was changed needed a second revision with change of the humeral component 25 days after. This was also the case in one of the patients with initial change of inlay who also needed a third intervention (conversion to a Hemi-prosthesis, 27 days later) followed by removal of the prosthesis (172 days after conversion) because of a chronic subluxation with compression of the brachial plexus and denial of the patient to undergo arthrodesis. Another patient also with initial change of inlay showed recurrent dislocation treated by closed reduction 91 days after followed by change of the prosthesis 171 days after.

One of the two patients who were revised because of a dislocation that could not be reduced closed, was treated with an exchange of the stem including correction of version. She developed an incomplete plexus palsy two days postoperatively which was attributed to tension, was treated conservatively with a splint maintaining the arm in flexion and recovered only partially.

### Infection and treatment procedure

In the cohort of 67 patients with 112 revisions, the total amount of indication for a revision was infection in ten cases, from which three were first revisions, six were second revisions and one third revision. Patients with infections were treated mostly with implantation of a spacer (n = 6) or with either debridement (n = 2) or total explantations of the prosthesis (n = 2). One of the patients was revised two times for infection. The most common pathogen was Propionibacterium acnes (n = 4). Broad antibiotic therapy was initiated intraoperatively and was replaced by antibiotics specific to the pathogen once results of bacteriological analyses were available and continued during a mean of 4 months (range 45 to 360 days). One of the patients with total explantation of the prosthesis died of an unrelated reason before 24 months follow-up after explantation. The outcome was not different in patients with revision for infection if compared with the whole cohort of 31 fully followed patients. The total Constant-Murley score of patients revised for infection increased also by 23 points (95% CI: 10.2, 37.6) from the time before RTSA to final follow-up as did the relative Constant-Murley score with a gain of 32% (95% CI: 24.7, 50.2) from a baseline that was very similar to the whole cohort (Table 4). In six cases there were no sings of a recurrent infection, in three cases the patients are still with an antibiotic spacer and do not wish to undergo re-implantation of a RTSA and one patient died of an unrelated cause with a resection arthroplasty for treatment of infection.

With the data available, we were unable to identify specific risk factors for infection in our material, specifically superficial hematoma and wound dehiscence were surprisingly not associated with a statistically higher infection rate.

## Discussion

Complications after RTSA are more common than after conventional total shoulder arthroplasty. The studies reporting these complications are heterogeneous in their methodology making a ranking of the most to the least common complication difficult [18]. More importantly, information about the relevance of the complications with regard to necessity for revision, as well as to the outcome after revision is sparse. The current study is retrospective and has therefore to be interpreted with caution. From a methodological point of view however, a prospective design for investigation of the relatively rare event of revision needed after RTSA would demand high resources and most probably a multicenter design. For the interpretation of the current data, incomplete documentation with inordinately high loss of follow-up might be a major problem. Lack of documentation was present in this study but only in the personal follow-up of 6 of the 37 patients. They had been reviewed clinically and radiographically in clinic at more than 24 months after their first revision but they were not scored according to Constant and Murley at that period of time. There were, however, no findings in the charts suggesting, that the scores or radiographic outcomes of these six patients would be markedly different from those of the other 31 patients. Another challenge for data interpretation was the multiplicity of indications for revision that could not be addressed by subgroup analysis because of the sample size.

The focus of this study was on the indications for RTSA revision in one specific department with a relatively large experience with RTSA rather than on an enumeration of the frequently discussed complications. In this respect, it is interesting that only one of 67 patients was revised because of inferior notching with contact of the inferior screw with the humeral component which had caused metallosis and pain but not loosening. Conversely there were other reasons that led not surprisingly to an overall high revision rate of 15% for RTSA. The cohort of the patients needing revision after RTSA consisted almost exclusively of those in whom RTSA has been implanted for salvaging situations not amenable to any alternative treatment and involving 100% of shoulders with previous operations (mean of 2 ± 1.4 operations). The revision rate of 15% documented in our cohort is inline with a recent report by Fevang et al.[20] of 36 revision needed for 225 RTSA (revision rate of 16%), in which the most common reason for revision after RTSA was identified to be aseptic loosening of the glenoid component followed by instability and dislocation. We identified instability as the most common reason for re-intervention after RTSA. This might be caused by the different indications of RTSA; the cohort investigated by Fevang et al.[20] consisted mainly of patients with inflammatory arthritis while the indication of RTSA in our cohort was mainly an irreparable massive rotator cuff with or without osteoarthritis.

We had started to use the Delta III reverse system in 1996 on a regular basis and changed to another system in 2005. For this study we attempted to eliminate the early phase of our learning curve and started our review with RTSAs which had been implanted no earlier than 1999. The overall revision rate was double as high with the first than with the second reverse system that we used. Although this difference is statistically significant we feel that it is mostly reflection of the fact that our knowledge, understanding and practical experience increased still substantially after 1999.

The most frequent indication for a re-intervention was instability and this for the first, the second and subsequent revisions. Almost all our operations were performed through a deltopectoral approach which has been identified as a risk factor for instability as compared with the superolateral approach [8]. For this problem, revision triggers have not changed during the study period, but the significantly lower revision rate with the second prosthetic design utilized may be related to better surgical technique and differences in indication. We cannot disagree but also not statistically affirm that early redislocation [8] can not be successfully treated with closed reduction alone and is probably a problem of component positioning including height of the prosthesis [21].

Hematoma and wound dehiscence have previously been identified as an inordinately frequent complication in our environment [12]. We currently still use two suction drains postoperatively for 48 hours in RTSA to drain the large subacromial space and have reduced postoperative hematoma and wound complications drastically. In revision surgery we will in addition to careful surgical hemostasis also use fibrin sealants to try to avoid hematoma formation and thereby the need for aspiration or revision. We feel that this complication is a truly surgical complication not in relation with the type of reverse implant utilized.

The 31 patients with a minimum follow-up of two years after the first revision who had undergone a mean of 1.8 revisions approximately doubled their Constant-Murley score from the time before RTSA to final follows-up. This was seen also in patients where the reason for revision was an infection. Although the range of motion increased in abduction and remained nearly the same in external rotation, it did not improve internal rotation. This observation has been described to be associated with RTSA [1] and not specific to patients with revision.

Except for instability, there were no relevant differences between the two prosthetic systems studied. The fact that the revision rate of the second system used was substantially lower was attributed to the increased knowledge concerning indication and increased skill in executing RTSA and may reflect the benefit of surgical experience in RTSA more so [22] than selecting one or the other system.

## Conclusions

Instability, hematoma or superficial wound complications and complications of the glenoid component are the most common reasons for an additional intervention after RTSA. Patients undergoing a revision as treatment of these complications profit significantly as long as the prosthesis remains in place.

## Competing interests

The senior author (GC) has received royalties from Zimmer Inc. The other authors did not receive anything of value from or own stock in a commercial company or institution related directly to the subject of this article.

## Authors’ contributions

All authors have made substantial contributions to this study; MF was involved in conception and design and analysis and interpretation of data and in drafting the manuscript. MG and SC were involved in the acquisition of data, interpretation of data and revising the manuscript critically for important intellectual content. GC was involved in conception and design of the study and interpretation of data, revising the manuscript critically for important intellectual content and supervision and coordination of the research group. All authors read and approved the final manuscript.

## Pre-publication history

The pre-publication history for this paper can be accessed here:

http://www.biomedcentral.com/1471-2474/13/160/prepub
